# Long-term outcomes after nucleos(t)ide analogues discontinuation in chronic hepatitis B patients with HBeAg-negative

**DOI:** 10.1186/1471-2334-13-458

**Published:** 2013-10-03

**Authors:** Dengming He, Shimin Guo, Wen Chen, Xianli Chen, Guohua Yan, Jie Wang, Maoshi Li, Peng Zhu, Hongfei Huang, Yuming Wang

**Affiliations:** 1 Institute of Infectious Diseases, Southwest Hospital, Third Military Medical University, Chongqing, China; 2 Liver Disease Diagnoses and Therapy Center, The 88th Hospital of the Chinese PLA, Tai'an, China; 3 Liver Disease Center, The 180th Hospital of the Chinese PLA, Quanzhou, China

**Keywords:** Chronic hepatitis B, HBeAg-negative, Nucleos(t)ide analogues, Discontinuation, Relapse

## Abstract

**Background:**

Hepatitis B e Antigen (HBeAg)-negative chronic hepatitis B (CHB) patients have an active liver disease with a high risk of progression to decompensated cirrhosis and hepatocellular carcinoma. The management strategy for HBeAg-negative CHB patients treated with nucleos(t)ide analogues (NUCs) is a topic of concern. To observe the outcomes for this population after NUCs withdrawal, HBeAg-negative CHB patients with loss of hepatitis B surface antigen (HBsAg) or sustained undetectable HBV DNA levels who had discontinued NUCs therapy were included in the study.

**Methods:**

A total of 66 patients (2 patients with HBsAg loss and 64 patients with sustained undetectable HBV DNA levels) were examined. HBV DNA levels and alanine aminotransferase (ALT) levels were monitored regularly after discontinuation of NUCs therapy. Relapse was defined as HBV DNA levels >2,000 IU/mL while off therapy in at least two determinations more than 4 weeks apart.

**Results:**

The time to achieve undetectable HBV DNA levels was 14 weeks (interquartile range (IQR): 12–24 weeks). The time until consolidation therapy was 144 weeks (IQR: 96–168 weeks). No relapses occurred in either of the HBsAg loss patients. Among the 64 patients with undetectable HBV DNA levels, 19 (29.7%) patients demonstrated evidence of relapse. All the relapses occurred within 96 weeks after discontinuation. The median duration of relapse was 36 weeks (IQR: 12–48 weeks). Elevation of HBV DNA and ALT levels over baseline was only observed in 10% of the relapse patients. There were no significant differences among the baseline characteristics (sex, HBV genotype, age, or ALT level) or the time until consolidation therapy between relapse and sustained-response patients.

**Conclusions:**

NUC discontinuation is feasible after achieving undetectable HBV DNA levels in HBeAg-negative CHB patients. Prolonging the time until consolidation therapy may be a good strategy to decrease the rate of relapse. More than 96 weeks of sustained response is a predictive marker of long-term sustained response.

## Background

About 350 million people worldwide live with chronic hepatitis B virus (HBV) infection. Approximately 25% of adults with chronic HBV infection since childhood later die from liver cancer or cirrhosis [1]. Therefore, the goal of chronic hepatitis B (CHB) therapy is to achieve sustained suppression of HBV replication and remission of liver disease. CHB may present either as hepatitis B e antigen (HBeAg)-positive or HBeAg-negative. The prevalence of HBeAg-negative CHB has been increasing over the last decade and represents the majority of cases in many areas. The strategy of long-term treatment with nucleos(t)ide analogues (NUCs) is necessary for HBeAg-positive patients [2]. Unlike interferons, which are administered for predefined durations of time owing to their immunomodulatory effects, NUCs are generally administered for a long duration, as long as 5–6 years or more, until specific end points are achieved, even though HBV DNA levels can be suppressed to undetectable in a much shorter period of time.

For HBeAg-negative patients, hepatitis B surface antigen (HBsAg) loss is the ideal endpoint [2]. However, previous longitudinal studies have indicated that the annual rate of HBsAg loss is only approximately 0.5–2.3% [3-6]. Induction of sustained off-therapy virological and biochemical response is a satisfactory end point [2]. However, relapse is frequent after NUCs discontinuation. Therefore, discontinuing or continuing therapy after achieving the satisfactory endpoint is a crucial decision faced by physicians and patients. Continuing therapy is typically the preferred choice for the majority of physicians because maintaining undetectable HBV DNA levels does not represent serum immunological response. Nevertheless, long-term therapy with NUCs may cause resistance or virological breakthrough.

Few studies have focused on the long-term outcomes after NUCs withdrawal in HBeAg-negative CHB patients. Based on ten years of data on NUCs therapy in HBeAg-negative CHB patients, we designed this retrospective study to observe the long-term outcomes of therapy discontinuation after achieving ideal or satisfactory endpoints.

## Methods

### Patients

HBeAg-negative CHB patients presenting for treatment at our hospital from 2002–2008, a total of 64 with sustained undetectable HBV DNA levels and 2 patients with HBsAg loss, were included in this study to observe the outcomes of therapy discontinuation. The patients who started out on one antiviral agent, such as lamivudine, and switched to another agent, such as entecavir, because they did not have a good response or developed resistance were classified by the last drug used. Patients were evaluated at least once every 3 months after discontinuation for ALT and HBV DNA levels. Clinical data were collected, monitored, and entered into a database. The outcomes, including sustained response and relapse, were evaluated during long-term follow-up for at least 24 weeks. Data were collected during follow-up appointments through January 1, 2012.

This analysis was conducted on anonymized data, collected as part of routine patient care. No additional investigations were performed. Therefore, no prior informed consent from the patients was required. The study protocol conformed to the ethical guidelines of the 1975 Declaration of Helsinki as reflected in a priori approval by the ethics committee of Southwest Hospital, which waived the need for informed consent.

### Inclusion and exclusion criteria

Criteria for inclusion included: presence of serum HBsAg for at least 6 months; negative for HBeAg; active CHB; and serum HBV DNA levels >2,000 IU/mL, as measured by LightCycler polymerase-chain-reaction (PCR) assay (Roche Molecular Diagnostics, Basel, Switzerland; lower limit of detection, 200 IU/mL). An active CHB was defined by a single serum alanine aminotransferase (ALT) level more than 2.0 times the upper limit of normal (ULN, 40 U/L) or at least two determinations 4 weeks apart of ALT levels 1.0–2.0×ULN.

Criteria for exclusion included: coexisting serious medical or psychiatric illness; organ or bone marrow transplantation; recent therapy with systemic corticosteroids, immunosuppressants, or chemotherapeutic agents; a serum alpha-fetoprotein level of at least 50 ng/mL; liver disease that was not due to hepatitis B; and seropositivity for human immunodeficiency virus or hepatitis C or D virus.

### Definitions of endpoints

In this study, two endpoints were considered: the ideal and the satisfactory. The ideal endpoint was sustained off-therapy HBsAg loss with or even without anti-HBs seroconversion. The satisfactory endpoint was sustained undetectable HBV DNA levels and biochemical response for at least 96 weeks.

### Definitions of outcomes after discontinuation

Outcomes after discontinuation were either 'sustained response’ or 'virological relapse’. Sustained response was defined as a sustained off-therapy virological and biochemical response. Virological relapse was defined as off-therapy HBV DNA levels >2,000 IU/mL in at least two determinations more than 4 weeks apart.

### Serological assays

Routine biochemical tests were performed using automated techniques. HBsAg, antibody to HBsAg, HBeAg, antibody to HBeAg, and antibody to hepatitis B core antigen were detected by ELISA (KHB, Shanghai, China) or electrochemiluminescence (Architect®, Abbott Laboratories, Abbott Park, IL, USA). Antibodies to hepatitis C virus, hepatitis D virus, and human immunodeficiency virus were detected by routine, commercially available enzyme immunoassays (KHB, Shanghai, China). Serum HBV DNA levels was quantified with a commercially available PCR assay (LightCycler®480 Real-Time PCR System, Roche). Alpha-fetoprotein levels were detected by routine, commercially available colorimetric assay (KHB). The HBV genotypes were determined using a hepatitis B virus gene type PCR fluorescence kit (Fosun, Shanghai, China).

### Statistical methods

Continuous variables were expressed as mean ± standard deviation (SD) for normal distributions or median and interquartile range (IQR) for abnormal distributions. Pearson’s chi-square tests, Kruskal-Wallis tests, Student’s t tests, ANOVAs, the Kaplan-Meier method, and log-rank tests were carried out as appropriate. All tests for significance and resulting *P* values were two-sided, with a level of significance of 0.05. The statistical software used for this analysis was SPSS 18.0.

## Results

### Baseline demographic and clinical characteristics

Baseline demographic and clinical characteristics of the patients are shown in Table 1. There were no significant differences in age, sex, ALT levels, or HBV DNA levels at baseline between patient’s with genotype B and genotype C. Baseline demographic and clinical characteristics of patient’s prescribed lamivudine, adefovir, entecavir, and telbivudine are shown in Table 2.

**Table 1:** Baseline demographic and clinical characteristics of patients

**Characteristic**	**Genotype B**	**Genotype C**	**P value**
Number - (%)	30 (45.5)	36 (54.5)	
Male - no. (%)	20 (66.7)	30 (83.3)	0.089
Age (Mean ± SD) - yr	36±13	35±10	0.191
<29 - no.	7	9	
30 - 39 - no.	15	20	
>39 - no.	8	7	
ALT [Median (IQR)] - ULN	2.6 (1.3-4.0)	3.2 (1.4-7.1)	0.219
<2×ULN - no.	13	14	
(2–5)×ULN - no.	13	8	
>5×ULN - no.	4	14	
HBV DNA (Mean±SD) - log10 IU/mL	5.6±1.4	5.6±1.5	0.908

**Table 2:** Baseline demographic and clinical characteristics of the NUCs discontinuation patients treated by lamivudine, adefovir, entecavir, and telbivudine

**Characteristic**	**LAM**	**ADV**	**ETV**	**LDT**
Number. (%)	15 (22.7)	42 (63.6)	7 (10.6)	2 (3.0)
Age (Mean ±SD) - yr	34.1±13.0	35.3±10.6	36.1±14.0	33.5±10.6
<29 - no.	4	9	2	1
30-39 - no.	8	24	3	0
>39 - no.	3	9	2	1
Man - no. (%)	14 (93.3)	30 (71.4)	4 (57.1)	2 (100)
ALT [Median (IQR)] - ULN	2.6 (1.2-3.5)	2.5 (1.3-6.6)	4.7 (2.7-14.0)	9.5 (3.9, 15.0)
HBV DNA - log10 IU/mL	6.3±1.6	5.3±1.3	5.5±0.8	7.0±2.2
HBsAg loss - no. (%)	2 (100)	0	0	0

*LAM*, lamivudine; *ADV*, adefovir; *ETV*, entecavir; *LDT*, telbivudine.

### Time of therapy

The time to achieve undetectable HBV DNA levels was 16 weeks (IQR: 12–24 weeks). The time of consolidation therapy was 144 weeks (IQR: 96–168 weeks). The total time of therapy was 160 weeks (IQR: 130–190 weeks). The time to achieve undetectable HBV DNA levels was 18 weeks (IQR: 12–24 weeks) with lamivudine, 16 weeks (IQR: 12–24 weeks) with adefovir, 8 weeks (IQR: 4–20 weeks) with entecavir, and 36 weeks (24 and 48 weeks) with telbivudine. The time of consolidation therapy was 192 weeks (IQR: 144–240 weeks) with lamivudine, 120 weeks (IQR: 96–144 weeks) with adefovir, 120 weeks (IQR: 96–144 weeks) with entecavir, and 96 weeks (96 and 96 weeks) with telbivudine. The time to achieve HBsAg loss in the two patients was 192 weeks and 288 weeks.

### Long-term outcomes after therapy discontinuation

Relapse was not observed in either of the two patients with HBsAg loss after 96 and 72 weeks. Therefore, the long-term outcomes after therapy discontinuation in this study focused on the patients with undetectable HBV DNA levels.

The time of follow-up in sustained response patients was 72 weeks (48–108 weeks). The proportion of relapse was 29.7% (19 of 64) and of sustained response was 70.3% (45 of 64). The distribution of outcomes after withdrawal of lamivudine, adefovir, entecavir, and telbivudine is shown in Table 3.

**Table 3:** Distribution of outcomes after discontinuation of lamivudine, adefovir, entecavir, and telbivudine

**NUCs**	**Sustained response - no. (%)**	**Relapse - no. (%)**
LAM	11 (24.4)	2 (10.5)
ADV	27 (60.0)	15 (78.9)
ETV	5 (11.1)	2 (10.5)
LDT	2 (4.4)	0

*LAM*, lamivudine; *ADV*, adefovir; *ETV*, entecavir; *LDT*, telbivudine.

To analyze the safety of relapse, ALT levels and HBV DNA levels were compared between baseline and the time of relapse. ALT levels higher than baseline at the time of relapse were only seen in 5.3% (1 of 19) of relapse patients. HBV DNA levels over baseline at the time of relapse were only seen in 10.5% (2 of 19) of relapse patients. No fulminant hepatitis was seen in any relapse patient. For patients with relapse, baseline and relapse levels of ALT and HBV DNA, as well as the treatment strategy after relapse, are shown in Table 4.

**Table 4:** Baseline and relapse levels of ALT and HBV DNA in patients with relapse

**No.**	**NUCs**	**HBV DNA - log10 IU/mL**		**ALT - ULN**		**Retreat**
		**Baseline**	**Relapse**	**Baseline**	**Relapse**	
1	LAM	7.70	3.39	1.67	1.02	ETV
2	LAM	7.67	3.51	3.88	1.45	ETV
3	ADV	3.91	3.35	1.43	0.57	ADV
4	ADV	4.14	3.74	2.71	0.74	ADV
5	ADV	5.88	3.41	1.48	0.36	ADV
6	ADV	5.42	5.71	7.33	4.90	ADV
7	ADV	7.71	3.41	8.00	0.83	ADV
8	ADV	3.87	3.44	1.26	1.00	ADV
9	ADV	6.32	3.43	1.31	0.98	ADV
10	ADV	5.39	3.77	1.05	0.36	ADV
11	ADV	5.86	4.06	6.69	1.21	ADV
12	ADV	3.33	3.39	1.38	0.90	ADV
13	ADV	6.37	3.60	5.07	1.31	ADV
14	ADV	5.68	4.51	6.57	1.57	ADV
15	ADV	4.42	4.15	1.08	0.98	ADV
16	ADV	4.68	3.31	1.32	0.17	ADV
17	ADV	7.39	4.06	3.55	0.60	ADV
18	ETV	4.55	5.22	2.74	1.07	ETV
19	ETV	5.63	3.68	1.19	1.29	ETV

*LAM*, lamivudine; *ADV*, adefovir; *ETV*, entecavir.

### Characteristics of patients with relapse

We further analyzed the baseline characteristics and therapy time of all patients to identify common features of the relapse patients. All relapse cases occurred within 96 weeks after therapy discontinuation. The time of relapse was 36 weeks (IQR: 12–48 weeks). No significant differences were noted in sustained response compared with relapse patients for time to undetectable HBV DNA levels, time of consolidation therapy, baseline HBV DNA levels, baseline ALT levels, baseline age, sex, or genotype (Table 5).

**Table 5:** Baseline characteristic and therapy time of relapse and sustained response (SR) patients after discontinuation

**Characteristic**	**SR (n=45)**	**Relapse (n=19)**	**P value**
HBV DNA - log10 IU/mL	5.5±1.5	5.5±1.5	0.928
ALT – × ULN	3.0 (1.5-6.3)	1.5 (1.2-5.1)	0.154
Age - years	34±11	37±11	0.310
Man - no. (%)	34 (75.6)	14 (73.7)	0.874
Time of achieving undetectable HBV DNA levels – weeks	12 (12–24)	16 (12–24)	0.412
Consolidation therapy - weeks	120 (96–156)	144 (96–144)	0.798
Genotype B - no. (%)	19 (65.5)	10 (34.5)	0.445
Genotype C - no. (%)	26 (74.3)	9 (25.7)	

### Cumulative sustained response rate of survival analysis

**Figure 1:**
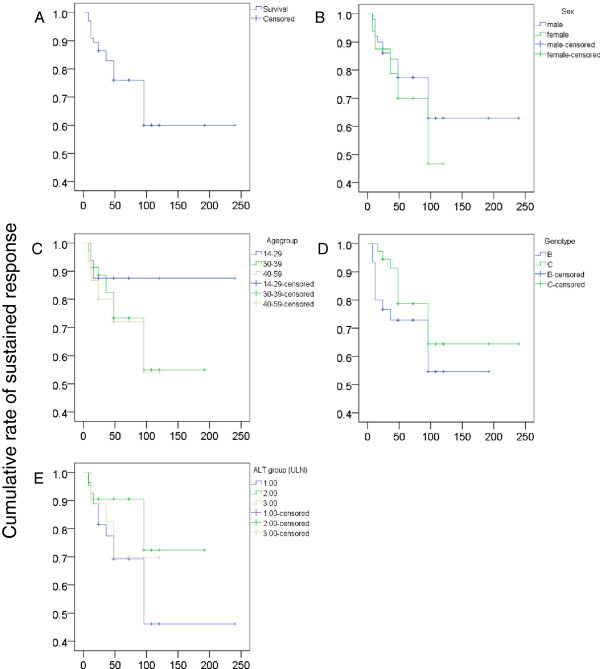
**Survival functions for the cumulative rate of sustained response and the time of sustained response after NUCs discontinuation.** The cumulative rate of sustained response was 71.2% **(A)**. No significant difference in the cumulative rate of sustained response was seen by sex (**B**, P=0.523), age group (**C**, P=0.445), genotype (**D**, P=0.322), or baseline ALT group (**E**, P=0.294).

To analyze the influence of multiple factors, we plotted survival functions between cumulative rate of sustained response and time of sustained response after NUCs discontinuation. The cumulative rate of sustained response was 71.2%. Although this analysis was performed using multiple factors, there were no significant differences in the cumulative rate of sustained response when comparing sex, age group, genotype, or baseline ALT group (Figure 1). Owing to the limited number of patients who were treated with telbivudine and entecavir, we did not perform survival analysis according to the different NUCs to avoid inappropriate or misleading results.

## Discussion

In the Mediterranean region and in Southeast Asia, 50 to 80% of CHB patients are HBeAg-negative [7]. HBeAg-negative CHB may follow seroconversion from HBeAg to anti-HBe antibodies during the immune reactive phase or may develop after years or decades in the inactive carrier state. HBV variations with nucleotide substitutions in the precore and/or the basal core promoter regions are unable to express, or express low levels, of HBeAg [8]. HBeAg-negative CHB patients have active liver disease with a high risk of progression to advanced hepatic fibrosis, cirrhosis, and subsequent complications including decompensated cirrhosis and hepatocellular carcinoma [8]. The strategy of long-term treatment with NUCs is necessary for HBeAg-negative CHB patients to prevent long-term sequelae. Although the use of NUCs has greatly improved HBeAg-negative CHB management, drug resistance is an important clinical risk resulting from long-term therapy. In this retrospective study, we observed the outcomes after therapy discontinuation in HBeAg-negative CHB patients after achieving HBsAg loss or sustained undetectable HBV DNA levels.

Relapse was not observed in patients who had achieved HBsAg loss after at least 96 weeks. Recent studies on HBsAg loss in HBeAg-negative patients demonstrated that relapse did not occur during the follow-up period [9,10]. This indicates that HBsAg loss is the preferred endpoint for therapy discontinuation. In another study, the prognosis after spontaneous HBsAg loss was good as long as cirrhosis did not emerge before HBsAg loss [11]. This result implies that HBsAg loss, therapy-induced or spontaneous, may be an ideal outcome for HBeAg-negative CHB patients. The inclusion criteria among these studies were very similar.

For HBeAg-negative CHB patients, relapse is frequent even when HBV DNA levels have been suppressed to undetectable for more than one year, making the precise endpoint for NUCs withdrawal unclear. The Asian Pacific Association for the Study of the Liver guidelines recommend that treatment can be discontinued if undetectable HBV DNA levels have been documented on three occasions ≥6 months apart. In the present study, consolidation therapy for at least 96 weeks was used as the satisfactory endpoint for NUCs discontinuation. The proportion of relapse after NUCs discontinuation was less than 30%, compared with 56% in a study with lamivudine [9], 61.4% in a 1-year study with adefovir [10], and 45.3% in a 1-year study with entecavir [12]. The relapse times we measured were longer than reported by other studies. On the one hand, the duration of consolidation therapy was longer than in other studies. A study showed that consolidation therapy for >64 weeks seems more appropriate for those with higher baseline HBV DNA [12]. On the other hand, short follow-up times for some patients may have influenced the outcomes.

Several studies of HBeAg-negative CHB patients after NUCs withdrawal showed that age was the only predictive factor for relapse, with lower relapse rates found in younger patients [9,10]. An HBV DNA decrease to <20,000 IU/mL at 12 weeks has been reported to be associated with a 50% chance of sustained off-treatment response [13]. A study of HBeAg-negative CHB patients after entecavir withdrawal showed that baseline HBV DNA ≤2×10^5^ IU/mL was the only significant independent factor predictive of a sustained response [12]. However, in the present study, there were no significant differences in cumulative sustained response rates among sex, baseline age group, baseline ALT levels, or genotype. This implies that there are no available routine clinical indicators to forecast relapse for HBeAg-negative CHB patients. However, according to our data, the factor most likely to be indicative of relapse or sustained response was baseline ALT levels. This result is actually consistent with the phenomenon of high ALT levels and high serum immunology response rates in HBeAg-positive CHB patients. For HBeAg-negative CHB patients, finding an appropriate indicator reflecting serum immunological response is necessary to determine the appropriate endpoint for NUCs discontinuation. Theoretically, in HBeAg-negative patients who achieved a sustained response after discontinuation, there must be a biomarker response indicative of the serum immunological response. The difference in anti-HBV immunity is essential, but there is currently no appropriate standard.

It is worth mentioning that all of relapses occurred within 96 weeks. This is consistent with the results published in previous studies [9,10,12], and implies that over 96 weeks of sustained response is a predictive marker for longer-term sustained response after NUCs discontinuation. Furthermore, elevated ALT levels and HBV DNA levels over baseline at relapse was observed in only 10% of relapse patients and no fulminant hepatitis was seen. This suggests that the safety risk of discontinuation is acceptable. In a recent study, the frequency of NUCs withdrawal flares was estimated as 3.2 per 100 person-years in 17 (11%) of 149 therapy discontinuations [14].

Two limitations in our data set should be noted. First, the follow-up time of some patients with sustained response was short (24 weeks in 7 patients, and 48 weeks in 15 patients). Second, the majority of patients used adefovir. The majority of NUCs prescribed in China from 2002–2008 were lamivudine and adefovir. Therefore, the number of patients who were treated with telbivudine and entecavir was very small. Based on the data from HBeAg-positive CHB patients treated with NUCs, the difference in serum immunology response rate among different drugs is very small. This suggests that sustained response depended on individual immunity to HBV rather than the different NUCs used.

## Conclusion

In conclusion, NUCs discontinuation is feasible after achieving undetectable HBV DNA in HBeAg-negative CHB patients. Prolonging the time of consolidation therapy after achieving undetectable HBV DNA may be a good strategy to decrease the rate of relapse. More than 96 weeks of sustained response is a predictive marker for long-term sustained response.

## Competing interests

The authors declare that they have no competing interests.

## Authors’ contributions

DH and WC performed the experiments. XC, GY, ML, SG, PZ, JW, and HH provided patients’ material and clinical data. Wang designed the study. DH and SG analyzed the data and wrote the paper. All authors read and approved the final manuscript.

## Pre-publication history

The pre-publication history for this paper can be accessed here:

http://www.biomedcentral.com/1471-2334/13/458/prepub
